# Book Reviews

**Published:** 1911-03

**Authors:** 


### BOOK REVIEWS

---

CEREBELLAR ABSCESS. By Heirich Neumann. Privat-Docent. University of Vienna. Translated by Richard Lake, F. R. C. S., London. H. K. Lewis, Gower Street. 156 pages.

This interesting and thorough piece of work consists of twelve chapters, of which the last, "Details of Cases," takes up half the volume. The book begins by an exhibition of statistics taken specially from reports by Koch, Okada, Korner, Heinemann and the author. Some of the interesting points therein brought out are that the intra-cranial abscess in the majority of cases appears between the tenth and thirtieth year of life, that labyrinthine suppuration in acute cases plays no role in the origination of the abscesses, but that in chronic cases disease of the labyrinth precedes the abscess.

Neumann also concludes that both cerebral and cerebellar abscess in 80 per cent. of cases originate from chronic suppuration of the middle ear, and that acute processes in the middle ear exhibit only a slight tendency to involvement of the endocranium. It is also noted that, as McEwan and Schwartze have shown, pus may exist in the middle ear without perforation of the membrane, as may also extensive disease of the mastoid, while the eburnation of the bone consequent upon prolonged diseases in the neighborhood may occur outwards while necrosis proceeds towards the brain.

Cholesteatoma plays an important part in producing cerebellar abscess by eroding the tissue and so providing a path of entrance for microbes. The abscess is usually of slow advance, weeks and months usually passing after its formation before the final catastrophe, which, however, occurs before the pus has formed in any great amount.

The author next proceeds to discuss the symptomatology, which he does fully and clearly, but into which we cannot here enter. Differential diagnosis and treatment are also fully covered, and in such a manner that we cannot do better than advise the reader to study the book for himself.

A. W. S.

---

A HANDBOOK OF PRACTICAL TREATMENT. In three volumes. By 79 eminent specialists. Edited by John H. Musser, M. D., Professor of Clinical Medicine, University of Pennsylvania, and A. O. Kelly, M. D., Assistant Professor of Medicine, University of Pennsylvania. Volume 1. Octavo of 909 pages. Illustrated. Philadelphia and London: W. B. Saunders Company, 1911. Per volume, cloth, $6.00 net; half morocco, $7.50 net.

This Handbook of Practical Treatment is one preeminently adapted to the practicing physician and gives the useful everyday observations of many well-known specialists. The forepart of the book is devoted to a discussion of the various therapeutic measures, while the latter part takes up the special treatment of general and local diseases. Special attention has been taken with organotherapy, serum therapy, bacteriotherapy, vaccine therapy and chemotherapy. It is from this part of the book that most useful and practical knowledge may be obtained; it is not so much in the administration of drugs that the average physician needs instruction, but to a much greater extent in the etiologic factors, dietetic and hygienic regulations, physiologic methods of treatment and early and opportune recourse to surgical intervention. In this part of the practice of medicine the volume under review seems superlative.

A chapter worthy of special attention is "The General Principles of Drug Treatment," by Sir Lander Brunton. Many of the others deserve mention—more than mention, they deserve careful reading and study by every physician who wishes to give to his patients the value of his fees.

SYSTEMIC (INCLUDING SPECIAL) PATHOLOGY. By J. George Adami, M. A., M. D., LL. D., F. R. S., Professor of Pathology, and Albert G. Nicholls, M. A., M. D., F. R. S., Assistant Professor of Pathology in McGill University, Montreal. In one octavo volume of 1,160 pages, with 301 engravings and 15 plates. Cloth $6.00 net. Lea & Febiger, Philadelphia and New York, 1911.

A little more than a year has sufficed to exhaust the first edition of this great work, but intead of resting satisfied with such evidence of approval, the authors have spared no effort in and thorough-going revision just published. This single volume covers the whole vast subject of speical pathology, and it would have been considerably smaller if it was not distinguished from other works by two very valuable features, namely, first, the inclusion of blood and circulatory organs (usually and erroneously considered under general pathology) and secondly, the treatment according to disturbances of function as well as of structure. Obviously, *function* and its derangements are what the clinician and practitioner most wish to understand, and structure is merely a step thereto. As a connecting link, therefore, between theory and practice, functional pathology is of prime importance, and a work properly emphasizing it is indispensable alike to the student and practitioner. Indeed, the keynote of the bok is to link theory to its practical application, and to be suggestive and helpful. Among the many changes effected in this revision, we may note that the chapters on the ductless glands and the nervous system have been rewritten, the latter being almost recreated. The section on the urinary system has been greatly amplified and the chapters on the circulatory and respiratory systems have been made more logical, coherent and adequate than before. In the new edition, Dr. Adami has written practically the whole of the introductions to the circulatory and respiratory sytems and the ductless glands and about half of that on the urinary function as well as sundry comments throughout the text, the remainder of the work being from the pen of Dr. Nicholls. In another sense, the whole of the volume represents both authors, as they have collaborated over its arrangement and harmoniously criticised and amended each other's work. It is risking little to predict that the second edition will increase the immense favor with which the original was received.

---

MODERN TREATMENT: THE MANAGEMENT OF DISEASE WITH MEDICINAL AND NON-MEDICINAL REMEDIES. By eminent American and English Authorities. Edited by Hobert Amory Hale, M. D., Professor of Therapeutics and Materia Medica, Jefferson Medical College, Philadelpaih; Physician to the Jefferson Hospital; Author of "A Text-Book of Practical Therapeutics," "A Text-Book of the Practice of Medicine," etc.; assisted by H. R. M. Landis, M. D., Medical Director to the Phipps Institute for Tuberculosis and Physician to the White Haven Sanitorium. In two very handsome, octavo volumes, comprising 1800 pages, with numerous engravings and full page plates. Price per volume in cloth, $6.00, net; half morocco, $7.50 net. Lea & Febiger, Publishers, Philadelphia and New York, 1911.

Scarcely two months have passed since the appearance of the first volume of "Modern Treatment," and now the work is completed by the issue of the second. Accordingly, it is fresh throughout, and justifies its title to the fullest extent, for every line is new and up-to-date, and the two volumes cover the whole field of present-day practical medicine in the most authoritative fashion. It is a good opportunity that is now offered to the profession to acquire the pith and essence of modern therapeusis in a form for instant reference. The work will find its natural place on the physician's desk rather than on his shelves, and is a handbook of helpful information for daily use rather than an encyclopedia.

